# Color Appearance of Various Provisional Restorative Materials for Rehabilitation Upon Aging

**DOI:** 10.1055/s-0042-1759886

**Published:** 2023-01-11

**Authors:** Niwut Juntavee, Apa Juntavee, Supichaya Srisontisuk

**Affiliations:** 1 Department of Prosthodontics, Faculty of Dentistry, Khon Kaen University, Khon Kaen, Thailand; 2 Division of Pediatric Dentistry, Department of Preventive Dentistry, Faculty of Dentistry, Khon Kaen University, Khon Kaen, Thailand; 3 Division of Biomaterials and Prosthodontics Research, Faculty of Dentistry, Khon Kaen University, Khon Kaen, Thailand

**Keywords:** CAD/CAM, color stability, provisional materials, polymer, resin, temporary restoration

## Abstract

**Objectives** Color stability of provisional restoration is crucial for full mouth reconstruction, which is probably influenced by aging. Therefore, this study evaluated the effect of aging on the color appearance of different provisional materials.

**Materials and Methods** Rectangular specimens (10 × 10 × 2 mm) were fabricated from computer-aided design-computer-aided manufacturing (CAD-CAM) (Vita CAD-Temp [VC], TelioCAD [TC], artBloc [RC]), autopolymerized (ProtempIV [PA], Luxatemp [LA], UnifastTrad [UA]), and heat-polymerized polymer (Major C&B [MH]). Each was divided into aging (AG, 5,000 thermocycles of 5°/55°C) and non-aging group ( *n* = 21/group). Color parameters were determined using a spectrophotometer through the CIELAB system. The L*, a*, and b* data were calculated for color appearance (∆ *E* ), translucency parameter (TP), contrast ratio (CR), and opalescence parameter (OP) for each.

**Statistical Analysis** An analysis of variance and multiple comparisons were determined for a significant difference in color appearance alteration of materials upon aging ( *α* = 0.05).

**Results** Statistically significant differences of L*, a*, b*, ∆ *E* , TP, CR, and OP due to the effect of types of material ( *p* < 0.05), aging effect ( *p* < 0.05), and their interactions ( *p* < 0.05) were indicated. Aging affects the color appearance, lightness, chromaticity, translucency, contrast, and opalescence of various types of provisional materials. Color appearance alteration of provisional materials was significantly indicated upon aging ( *p* < 0.05) but within the perceivable limit of clinical acceptance (∆ *E*_diff_ < 3.0), except for PA and MH. Color stability of CAD-CAM-based poly-methyl methacrylate (PMMA) and autopolymerized PMMA upon aging were better than bis-acryl resin and heat-polymerized PMMA. The TP, CR, and OP alterations of tested materials upon aging were within the perceivable limit.

**Conclusion** Color appearance of provisional restorative materials was influenced by aging. The CAD-CAM-based PMMA and autopolymerized PMMA materials showed better color stability than bis-acryl provisional resin and heat-polymerized PMMA upon aging. Therefore, they were suggested as provisional materials for oral rehabilitation.

## Introduction

Provisional restorations play a vital role in mimicking characteristics of definite restorations in fixed prosthetic treatment. They have been serving as a blueprint for extensive oral reconstructions, which significantly need aesthetic achievement, marginal accuracy, and functional durability throughout the treatment. 1 They must be capable of rendering aesthetic restorations, safeguarding the tooth abutment vitality, preventing tooth movement, and providing occlusal stability until the definitive restoration is cemented in the patient's mouth. The provisional restorations allow practitioners to determine the clinical appearance, patient satisfaction, phonetics, and appropriate vertical dimension. 2 They should be fabricated from a material that meets biological and esthetic requirements and possesses sufficient strength. Generally, provisional restorations are categorized into preformed and customized restorations. The preformed restorations are typically utilized for single restorations and are usually available in polycarbonate-, celluloid strip-, stainless steel-, or aluminum shell crowns. The temporary restorations for advanced oral rehabilitation are traditionally custom-made, either direct or indirect techniques, from various types of resins, including traditional autopolymerized (Ap-) or heat-polymerized (Hp-) acrylic-, bis-acryl-, and light-polymerized resin. 2 3 The resin acrylic in poly-methyl methacrylate (PMMA) or poly-ethyl methacrylate (PEMA) is frequently used. The PMMA-based provisional restoration is a noncrystalline microstructure containing a carboxyl group and methyl ester that offers excellent color stability and a well-defined margin. The PEMA-based temporary restoration comprises a smaller resin chain and is likely to provide less color stability and durability than PMMA-based restoration. 4 The bis-acryl provisional restoration mainly encompassed bis-phenyl glycidyl di-methacrylate in tightly connected microstructures. It is simply manageable, stable in color, and with slight contraction. 1 2 The light-cured provisional restoration comprising urethane-di-methacrylate demonstrates outstanding strength but an unfavorable delineated margin. 5 The contemporary evolution of computer-aided design-computer-aided manufacturing (CAD-CAM) technology has been implemented to construct temporary restoration employing subtractive or additive techniques. The subtractive technology utilizes the milling process of the prepolymerized PMMA block. 6 7 The additive technology uses the injectable process of a light-cured layering resin. The temporary restoration fabricated from the subtractive technique was exact compared to the additive strategies. 8 The prepolymerized PMMA promotes good homogeneity without polymerization shrinkage. Likewise, the temporary restoration from a subtractive technology offers a superior outcome to traditionally constructed or additively constructed temporary restoration regarding the stability of color, abrasion resistance, and uniformity of the restoration. 6 7 9 10 11

Dental clinicians generally select a provisional restorative material based on aesthetic color appearance, strength, marginal accuracy, ease of fabrication, and cost for fabrication of provisional restoration. 2 12 Among them, the color appearance is the most essential property, particularly for an extensive restoration that will be used for an extended period of treatment like oral reconstruction. In contrast, the stability of color appearance of provisional restoration is required. 13 14 In critically esthetic areas, it is a prime concern for temporary restoration, providing a primary esthetic shade matching the natural teeth and continuing color stability throughout its phase of service. Superlatively, temporary restorative material should maintain its color appearance after construction. Discoloration of provisional restoration may cause patient dissatisfaction, thus leading to surplus expenses for substitution. Discolorations are commonly classified as extrinsic discoloration from patient dietary and personal habits, for example, smoking, tobacco chewing, personal hygiene, plaque deposition and extrinsic stain, subsurface discoloration implying superficial degradation or absorption of stains within the superficial layer, and intrinsic discoloration from chemico-physical interactions in the interior portion of the provisional restoration. 15 Noticeable changes in the temporary restorative material's color may jeopardize the temporary restoration's acceptability. Color appearance of restoration involves light sources, receptors in human eyes, and material properties, whether it reflexes, transmits, or absorbs light. A spectrophotometer has been recommended to evaluate the color appearance because of its simplicity, practicality, and validity. The color can be measured using the Commission Internationale De L'éclairage (CIELAB) system via the optical parameters, including L* representing the lightness-darkness, a* designating the red-green, and b* defining the yellow-blue value of the color. The color appearance can be quantified in terms of translucency, contrast, opalescence, and color appearance difference of the restoration, that aimed at resemblance appearance to the natural tooth. 1 6 16 17

Provisional restorative polymer is likely to absorb liquid, rendering degradation at the covalent bonds in the polymeric chains, which could alter the color appearance of materials over time. 12 18 The resin matrix configuration, characteristics of fillers, polymerization mode, as well as aging also directly affect the susceptibility to color alteration of provisional material. 17 19 Previous studies stated that color sustainability of provisional resins is limited. 20 21 22 Aging of provisional material is a self-degrading process for a particular duration, which is associated with the biochemical compositions and performance behaviors of material to endure color alteration during function. 23 It was described that discoloration of resin composite is related to liquid penetration into the resin matrix and filler-matrix interface; thus, size and filler particles influenced aging discoloration. 24 25 26 Different methods have been used to bring out color alteration. However, the methods that evaluate the color appearance of the temporary restoration suddenly after finishing restoration or a short period of aging limit the capability to justify the clinical performance of the restorative material for oral reconstruction. Some investigations have used the immersion technique in solutions, for example, tea, coffee, juice, or others, for some time and compared their effects on the color alteration of materials. However, the type of immersion solution and period of immersion can significantly affect the amount of color alteration. 20 21 22 The accelerated thermocycle aging technique has been introduced to predict the clinically perceptible color change of material for long-term clinical experience. 20 21 22 27 The accelerated thermocycle aging provokes stress to the material and rushes up the deterioration effect on color change. 28 Previous studies suggested that some PMMA resins lean to undergo less discoloration over time than bis-acryl resins. In contrast, some studies have detected similarities in the color alteration of various provisional materials. 20 27 29 *Vis-a-vis* , a study described that provisional resin composite was not as color stable as provisional resin acrylic upon the simulated aging situation. 27 This suggests that the prediction of color stability of restorative material is still controversial regarding which provisional material performs superior color stability. Moreover, the investigation into the impact of aging on the color appearance of the CAD-CAM-formed and traditional-formed temporary restoration is absent. This investigation aimed to compare the effect of simulated aging on the color appearance of subtractively created CAD-CAM to traditional heat- and self-polymerized temporary restorative resins used for oral reconstruction. The null hypothesis stated no significant effect of simulated aging on the color appearance of different temporary materials.

## Materials and Methods

The *in vitro* investigation was performed with sample size estimation from the former study 2 using the Pi-face program version 1.76 with 90% power of the test, two-tailed analysis, and 0.05 level of significance.

### Specimen Preparation

Two hundred and ninety-four (294) specimens (10 × 10 × 2 mm) were prepared according to International Organization for Standardization no. 10477-2004 from two prepolymerized PMMA-CAD-CAM blanks (Vita CAD Temp [VC], Telio CAD [TC]), one acrylate polymer CAD-CAM blank (artBloc [RC]), two autopolymerized bis-acryl resin composites (Protemp IV [PA], Luxatemp star [LA]), one autopolymerized PMMA resin (Unifast Trad [UA]), and one heat-polymerized PMMA resin (Major C&B [MH]) as shown in Table 1 . The VC, TC, and RC CAD-CAM blocks were segmented with a precision cutting instrument (Isomet-1000, Buehler, Lake Bluff, Illinois, United States). The PA and LA bis-acryl resin specimens were constructed by injectable resins into the lubricated stainless steel mold and left for complete polymerization. Then the sample was recovered. The UA autopolymerized PMMA samples were fabricated from the resin polymer mixture at a powder–polymer ratio to liquid monomer equal to 1:2, compressed with 2.5 psi pressure in a stainless steel mold for 5 minutes at room temperature. The MH heat-cured PMMA samples were fabricated from the resin polymer mixture at a powder–polymer ratio to a liquid monomer of 1:2, pressed in a gypsum mold, and cured at 70°C for 8 hours. The specimens were finished and smoothed with 1,200 grits abrasives in the finishing instrument (Ecomet-3, Buehler). One investigator prepared all samples and submersed them in 37°C of artificial saliva (pH = 6.73) for 24 hours before randomly allocating each type of sample into nonaging (NG) and artificial aging (AG) groups. The samples in the AG groups were exposed to thermocycle aging. The samples in the NG group were left untreated and used as control.

**Table 1:** Compositions, types, polymerization process, manufacturers, and batch number of provisional materials used in this study

Materials	Group	Manufacturer	Batch no.	Shade	Compositions	Types	Polymerization
Vita CAD Temp	VC	Vita Zahnfabrik, Bad Sackinken, Germany	48000	3M2	Poly-methyl methacrylate Acrylate polymer Microfiller reinforced poly-acrylic	Acrylate polymer	CAD/CAM
Telio CAD	TC	Ivoclar Vivadent, Bensheim, Germany	WV7938	A3	Poly-methyl methacrylate	PMMA	CAD/CAM
artBloc	RC	Merz Dental GmbH, Lutienburg, Germany	14916	A3	Poly-methyl methacrylate	PMMA	CAD/CAM
Protemp IV	PA	3M ESPE, MN, USA	B642333	A3	Dimethacrylate (BISEMA6) Silane-treated amorphous silica Reaction products of 1,6- diisocyanatohexane with 2-[(2-methacryloyl)ethyl]6- hydroxyhexanoate 2-Hydroxyethyl Methacrylate (DESMA)	Bis-acryl resin	Auto
Luxatemp star	LA	Zenith-DMG/ Foremost Dental, NJ, USA.	755605	A3	Urethane dimethacrylate Aromatic dimethacrylate Glycol methacrylate (cross-linking agent) Glass powder Silica	Bis-acryl resins	Auto
Unifast Trad	UA	GC Corp., Tokyo, Japan	1311221	Ivory	Methyl methacrylate (MMA) (Liquid) Thyl and Ethyl methacrylate copolymer Poly(methyl methacrylate) Di-isopropyl 3,3′-[(2,5-dichloro-1,4-phenylene) bis[iminocarbonyl(2-hydroxy-3,1-naphthylene)azol] bis[4 methyl benzoate]	PMMA	Auto
Major C&B-V	MH	Major Prodotti Dentari S.P.A., Moncalieri, Italy	011110	4F	Methyl methacrylate	PMMA	Heat

Abbreviations: CAD/CAM, computer-aided design/computer-aided manufacturing; PMMA, poly-methyl methacrylate.

### Aging Process

The thermocycling procedure accomplished the aging process by immersion of the AG samples in a tempering bath comprising a cold (5°C) water bath and a (55°C) warm water bath. Each specimen group was organized in color-coded vessels and alternatively immersed for 30 seconds in the warm and cold water baths with 10 seconds of transferring time between each bath. The thermocycles were performed for 5,000 cycles.

### Evaluation of Color Appearance

The color appearance of each specimen was evaluated using a spectrophotometer (ColorQuest XE, Hunter, Reston, Virginia, United States). The machine was set for a 2-degree angle of observer, a 4-mm diameter of an aperture, 100% ultraviolet, and D65 illuminant as a standard 380 to 780 nm wavelength. Before carrying out the measurements, the machine was standardized with a standardized white tile. To eliminate the edge loss effect and remain in a precise position during optical measurement, a transparent jig was utilized for positioning the sample during determining color parameters measuring in three exact places; Left-center-right. First, the CIELAB color space was determined by L* (lightness), a* (green-red), and b* (blue-yellow) coordinates for each sample. Next, the data were computed for color appearance (∆ *E* ), translucency parameter (TP), contrast ratio (CR), and opalescence parameter (OP) for each group. 30 The color appearance against a white background (∆ *E _w_* ) was calculated according to Eq. (1) .



The translucency was determined from TP by computing the color differences between standard black [( *B* ), *L* * =10.4, *a* * = 0.4, *b* * = 0.6] and standard white [( *W* ), *L* * = 96.7, *a* * = 0.1, *b* * = 0.2] backgrounds as Eq. (2) .



The luminance upon tristimulus color space or spectrum reflectance [ *Y* ] was computed from L* values, as shown in Eq. (3) . The specified white stimulus signified a complete reflecting diffuser and normalized by a common factor to derive for *Y _n_* that equaled 100. 30 Finally, the *Y* value of the sample measured upon white ( *Y*_W_ ) and black ( *Y*_B_ ) background was used to assess CR as Eq. (4) .





The opalescence was determined from OP by computing the value a* and b* color coordinate difference between the white (W) and black (B) backgrounds as Eq. (5) .



The color appearance difference (∆ *E*_diff_ ) was computed from the differences in L*, a*, and b* coordinates upon a white background between the NG and AG process using Eq. (6) .



### Microscopic Examination

The specimen was cleaned with distilled water in the ultrasonic cleaner, dried with the desiccator (Northman, Hsein, Taiwan), and sputter-coated with gold-palladium using 10 mA current and130 mTorr vacuum for 3 minutes in a coating machine (K500X Emitech, Ashford, England). Then, the microscopic scanned surfaces were examined with the scanning electron microscope (SEM; Hitachi S-3000N, Osaka, Japan).

### Statistical Analysis

The data were assessed for normality using the Kolmogorov–Smirnov test. The two-way analysis of variance (ANOVA) was performed to validate for significant differences ( *p* < 0.05) for color parameters using SPSS/PC V. 22 software (IBM, Armonk, New York, United States). Determination of substantial differences among groups was tested using Bonferroni post hoc comparisons.

## Results

**Fig. 1:**
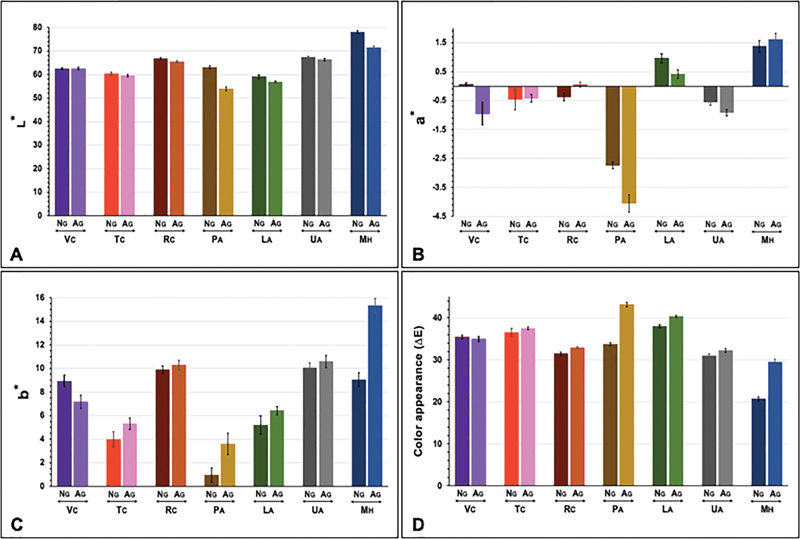
Color parameter L* ( **A** ), a* ( **B** ), b* ( **C** ), and color appearance ( **D** ) of provisional materials, including computer-aided design-computer-aided manufacturing (CAD-CAM) (Vita CAD Temp [VC], Telio CAD [TC], artBloc [RC]), auto- (Protemp IV [PA], Luxatemp star [LA], Unifast Trad [UA]), and heat-polymerized poly-methyl methacrylate resin (Major C&B-V [MH]) upon aging (AG) versus nonaging (NG).

**Fig. 2:**
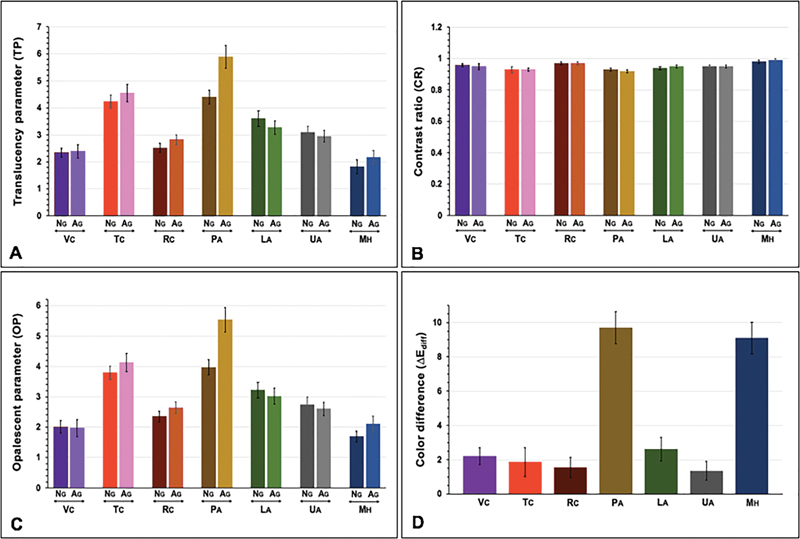
Translucency parameter ( **A** ), contrast ratio ( **B** ), opalescence parameter ( **C** ), and color difference ( **D** ) of provisional materials, including computer-aided design-computer-aided manufacturing (CAD-CAM) (Vita CAD Temp [VC], Telio CAD [TC], artBloc [RC]), auto- (Protemp IV [PA], Luxatemp star [LA], Unifast Trad [UA]) and heat-polymerized poly-methyl methacrylate resin (Major ^®^ C&B-V [MH]) upon aging (AG) versus nonaging (NG).

The mean, standard deviation, 95% confidential interval of L*, a*, b*, ∆ *E* , TP, CR, and OP upon NG and AG, and ∆ *E*_diff_ are presented in Table 2 , Fig. 1 , and Fig. 2 , respectively. ANOVA indicated a statistically significant difference of L*, a*, b*, ∆ *E* , TP, CR, and OP owing to the effect of types of material ( *p* < 0.05), aging effect ( *p* < 0.05), and their interactions ( *p* < 0.05) as shown in Table 3 . In addition, aging significantly affected ∆ *E*_diff_ ( *p* < 0.05), as shown in Table 3 . The pairwise post hoc multiple comparisons represented a significant effect of the type of materials on color parameters, shown in Table 4 . The statistics suggested significant differences in L* among types of material ( *p* < 0.05), except for TC-RC-UA ( *p* > 0.05). The statistics revealed significant differences of a* among types of material ( *p* < 0.05), except for groups of VC-PA, TC-MH, and RC-MH ( *p* > 0.05). The statistics indicated significant differences of b* among types of material ( *p* < 0.05), except for groups of TC-LA and RC-UA ( *p* > 0.05). The statistics indicated significant differences in ∆ *E* and TP among all types of material ( *p* < 0.05), as shown in Table 4 . The statistics indicated significant differences in CR among types of material ( *p* < 0.05), except for groups of VC-TC-RC-UA and groups of PA-TC and PA-MH ( *p* > 0.05). The statistics indicated significant differences in OP among types of material ( *p* < 0.05), except for UA-RC ( *p* > 0.05). The statistics indicated significant differences in ∆ *E*_diff_ among types of material ( *p* < 0.05), except for VC-TC-RC-LA, TC-RC-UA, RC-UA, and PA-MH ( *p* > 0.05).

**Table 2:** Mean, standard deviation (SD), 95% confidential interval (CI) of lightness value (L*), red-green coordinate (a*), yellow-blue coordinate (b*), color appearance (Δ *Ε* ), translucency parameter (TP), contrast ratio (CR), opalescent parameter (OP), and color appearance difference (Δ *Ε*_diff_ ) of different provisional materials including CAD-CAM (Vita CAD Temp [VC], Telio CAD [TC], artBloc [RC]), auto- (Protemp IV [PA], Luxatemp star [LA], Unifast Trad [UA]), and heat-polymerized poly-methyl methacrylate resin (Major C&B-V [MH]) upon aging (AG) versus nonaging (NG)

Group	*n*	L*	a*	b*	Δ *Ε*	TP	CR	OP	Δ *Ε*_diff_
		Mean ± SD (95% CI)	Mean ± SD (95% CI)	Mean ± SD (95% CI)	Mean ± SD (95% CI)	Mean ± SD (95% CI)	Mean ± SD (95% CI)	Mean ± SD (95% CI)	Mean ± SD (95% CI)
VCNG	21	62.39 ± 0.51 (62.16–62.62)	0.07 ± 0.06 (0.01–0.13)	8.93 ± 0.48 (8.71–9.15)	35.41 ± 0.51 (35.18–35.64)	2.34 ± 0.17 (2.26–2.42)	0.96 ± 0.01 (0.95–0.96)	2.01 ± 0.2 (1.92–2.10)	2.21 ± 0.49 (1.99–2.44)
VCAG	21	62.46 ± 0.57 (62.20–62.73)	–0.95 ± 0.40 (–1.14)–(–1.77)	7.17 ± 0.72 (6.84–7.50)	34.96 ± 0.63 (34.68–35.25)	2.39 ± 0.25 (2.28–2.51)	0.95 ± 0.02 (0.94–0.96)	1.97 ± 0.28 (1.84–2.09)	
TCNG	21	60.39 ± 0.65 (60.09–60.68)	–0.45 ± 0.37 (–0.70)–(–0.19)	3.99 ± 0.94 (3.56–4.42)	36.53 ± 0.64 (36.24–36.82)	4.24 ± 0.24 (4.12–4.35)	0.93 ± 0.02 (0.92–0.94)	3.79 ± 0.21 (3.69–3.88)	1.86 ± 0.84 (1.48–2.25)
TCAG	21	59.58 ± 0.48 (59.36–59.80)	–0.42 ± 0.14 (–0.49)–(–0.36)	5.32 ± 0.30 (5.19–5.46)	37.48 ± 0.50 (37.25–37.71)	4.55 ± 0.32 (4.40–4.70)	0.93 ± 0.01 (0.92–0.94)	4.13 ± 0.30 (4.00–4.27)	
RCNG	21	66.76 ± 0.34 (66.60–66.91)	–0.37 ± 0.13 (–0.43)–(–0.32)	9.88 ± 0.38 (9.70–10.05)	31.47 ± 0.40 (31.29–31.66)	2.52 ± 0.17 (2.44–2.60)	0.97 ± 0.01 (0.96–0.98)	2.35 ± 0.18 (2.27–2.44)	1.55 ± 0.58 (1.29–1.82)
RCAG	21	65.38 ± 0.41 (65.19–65.57)	0.05 ± 0.09 (–0.04)–(0.15)	10.27 ± 0.18 (10.19–10.35)	32.90 ± 0.38 (32.73–33.08)	2.82 ± 0.18 (2.74–2.90)	0.97 ± 0.01 (0.96–0.98)	2.64 ± 0.19 (2.56–2.73)	
PANG	21	63.10 ± 0.60 (62.83–63.37)	–2.74 ± 0.12 (–2.80)–(–2.68)	0.95 ± 0.39 (0.78–1.13)	33.73 ± 0.61 (33.46–34.01)	4.40 ± 0.26 (4.28–4.52)	0.93 ± 0.01 (0.92–0.94)	3.97 ± 0.25 (3.86–4.09)	9.70 ± 0.94 (9.27–10.13)
PAAG	21	53.87 ± 0.91 (53.45–54.29)	–4.05 ± 0.30 (–4.19)–(–3.92)	3.59 ± 0.52 (3.36–3.83)	43.17 ± 0.92 (42.75–43.59)	5.89 ± 0.43 (5.70–6.09)	0.92 ± 0.01 (0.91–0.93)	5.54 ± 0.40 (5.35–5.72)	
LANG	21	59.07 ± 0.77 (58.72–59.42)	0.96 ± 0.16 (0.88–1.03)	5.21 ± 0.33 (5.07–5.36)	37.97 ± 0.79 (37.61–38.33)	3.60 ± 0.29 (3.47–3.73)	0.94 ± 0.01 (0.93–0.95)	3.22 ± 0.26 (3.10–3.33)	2.61 ± 0.69 (2.30–2.92)
LAAG	21	56.86 ± 0.35 (56.70–57.02)	0.42 ± 0.15 (0.35–0.49)	6.43 ± 0.24 (6.33–6.54)	40.32 ± 0.36 (40.16–40.49)	3.27 ± 0.25 (3.15–3.38)	0.95 ± 0.01 (0.94–0.96)	3.02 ± 0.26 (2.90–3.11)	
UANG	21	67.36 ± 0.44 (67.16–67.57)	–0.55 ± 0.12 (–0.61)–(–0.50)	10.06 ± 0.39 (9.88–10.23)	30.96 ± 0.46 (30.75–31.16)	3.10 ± 0.22 (3.00–3.20)	0.95 ± 0.01 (0.94–0.96)	2.73 ± 0.27 (2.60–2.85)	1.35 ± 0.55 (1.09–1.60)
UAAG	21	66.23 ± 0.52 (65.99–66.46)	–0.91 ± 0.11 (–0.96)–(–0.86)	10.59 ± 0.35 (10.43–10.75)	32.21 ± 0.51 (31.98–33.44)	2.95 ± 0.22 (2.85–3.05)	0.95 ± 0.01 (0.94–0.96)	2.60 ± 0.22 (2.50–2.70)	
MHNG	21	78.02 ± 0.59 (77.78–78.29)	1.37 ± 0.20 (1.28–1.46)	9.05 ± 0.36 (8.89–9.22)	20.72 ± 0.53 (20.48–20.96)	1.82 ± 0.25 (1.71–1.94)	0.98 ± 0.01 (0.97–0.99)	1.69 ± 0.18 (1.66–1.77)	9.09 ± 0.92 (8.67–9.51)
MHAG	21	71.46 ± 0.60 (71.19–71.73)	1.61 ± 0.22 (1.51–1.71)	15.31 ± 0.85 (14.92–15.70)	29.47 ± 0.71 (29.15–29.79)	2.17 ± 0.25 (2.06–2.29)	0.99 ± 0.01 (0.98–1.0)	2.10 ± 0.27 (1.98–2.22)	

**Table 3:** An analysis of variance (ANOVA) of lightness value (L*), red-green coordinate (a*), yellow-blue coordinate (b*), color appearance (Δ *Ε* ), translucency parameter (TP), contrast ratio (CR), opalescent parameter (OP), and color appearance difference (Δ *Ε*_diff_ ) of different provisional materials of different provisional materials upon aging versus nonaging (NG)

ANOVA of lightness value (L*) of monolithic ceramic materials upon different tempering processes						
**Source**		**SS**	**df**	**MS**	***F***	***p***
Aging	Greenhouse-Geisser	677.22	1	677.22	1391.963	0.001
Material	Greenhouse-Geisser	8925.51	4.327	2062.92	3975.43	0.001
Aging*Material	Greenhouse-Geisser	760.95	4.459	170.67	494.61	0.001
ANOVA of red-green coordinate (a*) of monolithic ceramic materials upon different tempering processes						
**Source**		**SS**	**df**	**MS**	***F***	***p***
Aging	Greenhouse-Geisser	9.739	1	9.739	111.376	0.001
Material	Greenhouse-Geisser	583.856	3.199	182.534	1572.548	0.001
Aging*Material	Greenhouse-Geisser	26.232	2.434	10.738	74.857	0.001
ANOVA of yellow-blue coordinate (b*) of monolithic ceramic materials upon different tempering processes						
**Source**		**SS**	**df**	**MS**	**F**	***p***
Aging	Greenhouse-Geisser	168.979	1	168.979	1356.389	0.001
Material	Greenhouse-Geisser	3146.842	3.158	996.332	1353.938	0.001
Aging*Material	Greenhouse-Geisser	386.483	2.503	154.409	475.475	0.001
**ANOVA of color appearance (Δ** ***Ε*** **) of monolithic ceramic materials upon different tempering processes**						
**Source**		**SS**	**df**	**MS**	***F***	***p***
Aging	Greenhouse-Geisser	844.47	1	844.47	1462.00	0.001
Material	Greenhouse-Geisser	6095.44	4.09	1490.44	2644.13	0.001
Aging*Material	Greenhouse-Geisser	1002.26	4.20	238.35	632.44	0.001
**ANOVA of translucency parameter (TP) of monolithic ceramic materials upon different tempering processes**						
**Source**		**SS**	**df**	**MS**	***F***	***p***
Aging	Greenhouse-Geisser	6.13	1	6.13	232.60	0.001
Material	Greenhouse-Geisser	321.47	3.98	80.75	612.27	0.001
Aging*Material	Greenhouse-Geisser	21.98	3.94	5.57	103.35	0.001
**ANOVA of contrast ratio (CR) of monolithic ceramic materials upon different tempering processes**						
**Source**		**SS**	**df**	**MS**	***F***	***p***
Aging	Greenhouse-Geisser	0.00	1	0.00	39.94	0.001
Material	Greenhouse-Geisser	0.05	4.03	0.01	45.94	0.001
Aging*Material	Greenhouse-Geisser	0.06	4.36	0.01	53.71	0.001
**ANOVA of opalescent parameter (OP) of monolithic ceramic materials upon different tempering processes**						
**Source**		**SS**	**df**	**MS**	***F***	***p***
Aging	Greenhouse-Geisser	0.00	1	0.00	0.94	0.001
Material	Greenhouse-Geisser	71.63	4.92	14.55	221.33	0.001
Aging*Material	Greenhouse-Geisser	236.47	4.83	48.94	667.47	0.001
** ANOVA of color appearance difference (Δ *Ε*_diff_ ) of monolithic ceramic materials upon different tempering processes **						
**Source**		**SS**	**df**	**MS**	***F***	***p***
Between group		1703.05	6	283.842	520.177	0.001
Within group		76.393	140	0.564		
Total		1779.442	146			

Abbreviations: df, degree of freedom; *F* , *F* -ratio; MS, mean square; SS, sum of squares.

**Table 4:** Post hoc Bonferroni multiple comparisons of lightness value (L*), red-green coordinate (a*), yellow-blue coordinate (b*), color appearance (Δ *Ε* ), translucency parameter (TP), contrast ratio (CR), opalescent parameter (OP), and color appearance difference (Δ *Ε*_diff_ ) of different provisional materials of different provisional materials upon aging versus nonaging (NG)

Post hoc multiple comparison of lightness (L*)								Post hoc multiple comparison of lightness (a*)						
	VC	TC	RC	PA	LA	UA	MH	VC	TC	RC	PA	LA	UA	MH
VC	1	0.005	0.001	0.001	0.001	0.001	0.001	1	0.001	0.001	0.174	0.001	0.001	0.001
TC		1	0.351	0.001	0.001	1	0.001		1	0.007	0.001	0.001	0.012	1
RC			1	0.001	0.012	1	0.001			1	0.001	0.001	0.001	1
PA				1	0.001	0.001	0.001				1	0.001	0.001	0.001
LA					1	0.001	0.001					1	1	0.001
UA						1	0.001						1	0.001
MH							1							1
Post hoc multiple comparison of lightness (b*)								**Post hoc multiple comparison of color appearance (Δ** ***Ε*** **)**						
VC	1	0.001	0.001	0.001	0.001	0.001	0.001	1	0.001	0.001	0.001	0.001	0.001	0.001
TC		1	0.001	0.001	1	0.001	0.001		1	0.001	0.001	0.001	0.001	0.001
RC			1	0.001	0.001	1	0.001			1	0.001	0.001	0.001	0.001
PA				1	0.001	0.001	0.001				1	0.024	0.001	0.001
LA					1	0.001	0.001					1	0.001	0.001
UA						1	0.001						1	0.001
MH							1							1
**Post hoc multiple comparison of translucency parameter (TP)**								**Post hoc multiple comparison of contrast ratio (CR)**						
VC	1	0.001	0.001	0.001	0.001	0.001	0.001	1	0.340	1	0.001	0.001	1	0.001
TC		1	0.001	0.001	0.001	0.001	0.001		1	1	0.170	0.001	1	0.030
RC			1	0.001	0.001	0.001	0.001			1	0.010	0.001	1	0.001
PA				1	0.000	0.001	0.001				1	0.001	0.001	1
LA					1	0.001	0.001					1	0.001	0.001
UA						1	0.001						1	0.001
MH							1							1
**Post hoc multiple comparison of opalescent parameter (OP)**								** Post hoc multiple comparison of Δ *Ε*_diff_**						
VC	1	0.001	0.001	0.001	0.001	0.001	0.001	1	1	0.091	0.001	1	1	0.004
TC		1	0.001	0.001	0.001	0.001	0.001		1	1	0.001	0.029	0.514	0.001
RC			1	0.001	0.001	0.794	0.001			1	0.001	0.001	1	0.001
PA				1	0.001	0.001	0.001				1	0.001	0.001	0.168
LA					1	0.001	0.001					1	0.001	0.001
UA						1	0.001						1	0.001
MH							1							1

The translucency of material indicated by TP was significantly affected by aging ( *p* < 0.05). The translucency was increased considerably upon aging for VC, TC, RC, PA, and MH ( *p* < 0.05). Vice versa, translucency was significantly reduced upon aging for LA and UA ( *p* < 0.05). As indicated by CR, the contrast of material was affected considerably by aging ( *p* < 0.05). The contrast was significantly decreased upon aging for VC, TC, RC, PA, and MH ( *p* < 0.05). Vice versa, the contrast was increased considerably upon aging for LA and UA ( *p* < 0.05). As indicated by OP, the opalescence of material was significantly affected by aging ( *p* < 0.05). The opalescence was increased considerably upon aging for TC, RC, PA, and MH ( *p* < 0.05). Vice versa, translucency was significantly decreased upon aging for VC, LA, and UA ( *p* < 0.05). The amount of color alteration of different types of material upon aging, as indicated from the highest to the lowest ∆ *E*_diff_ value, were PA (9.70), MH (9.09), LA (2.61), VC (2.21), TC (1.86), RC (1.55), and UA (1.35), respectively.

**Fig. 3:**
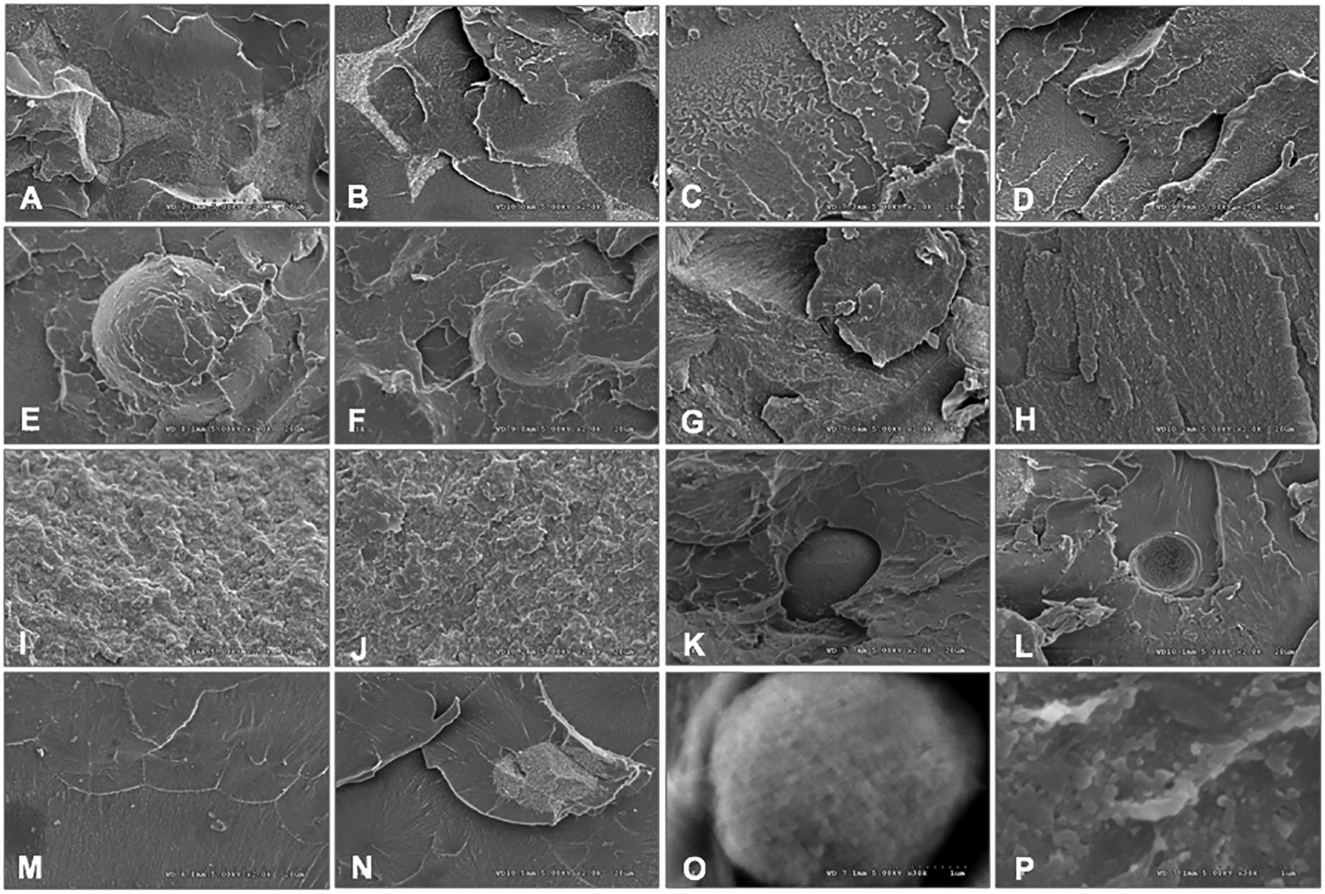
Scanning electron microscope (SEM) topographic photomicrographs (X2K) of provisional materials including computer-aided design-computer-aided manufacturing (CAD-CAM) (Vita CAD Temp ( **A** , **B** ), Telio CAD ( **C** , **D** ), artBloc ( **E** , **F** )), auto- (Protemp IV ( **G** , **H** ), Luxatemp star ( **I** , **J** ), Unifast Trad ( **K** , **L** )), and heat-polymerized poly-methyl methacrylate resin (Major C&B-V ( **M** , **N** ) upon nonaging ( **A** , **C** , **E** , **G** , **I** , **K** , **M** ) versus aging ( **B** , **D** , **F** , **H** , **J** , **L** , **N** ). Significant void defect in auto- ( **O** ) and less defect in heat- ( **P** ) polymerized poly-methyl methacrylate resin at X30KX were indicated.

The SEM photomicrographs disclosed differences in material characteristics for both nonaged ( Fig. 3A , C, E, G, I, K, M, O) and aged groups ( Fig. 3B , D, F, H, J, L, N, P) as a consequence of the different microstructure of materials. The VCAG and VCNG indicated prominent microfillers reinforcing poly-acrylic surrounding acrylate polymer. Little granules deposited on surfaces were revealed. The VCNG was denser than VCAG. The TCAG and TCNG displayed different coarseness contained with a dense and smooth resin matrix. The TCNG microstructure indicated a constant linear configuration, whereas TCAG exhibited a mixed pattern microstructure. The RCAG and RCNG revealed an analogous arrangement to the TCAG and TCNG group, with minimal granule implanting in the resin matrix. The PAAG and PANG revealed both smooth and irregular surfaces. The LAAG and LANG demonstrated rough surface configuration. The UAAG and UANG disclosed a comparable surface configuration to the RCAG and RCNG group, with a distinct rugged surface configuration beside minute voids included in the matrix of resin. The MHAG and MHNG presented dense microstructure with some areas of roughness. The micrograph indicated a significant void defect in auto- ( Fig. 3O ) and less defect in heat- ( Fig. 3P ) polymerized PMMA at X30K.

## Discussion

Promising the color stability of provisional restoration is a fundamental factor with a powerful impact on the successful treatment of oral reconstruction. In consideration of the phase of provisional temporalization, patients are always concerned about the aesthetics and function of temporary restorations. The color alteration may lead to aesthetic complications when provisional restorations are planned for temporalization for a long time. Color alteration of provisional restorations may associate with the patient's diet, personal hygiene, and personal habits such as smoking, tobacco chewing, and material properties. This study indicated that different types of provisional material and the aging process influenced the color appearance of provisional materials tested. Aging is affected by different color parameters, including L*, a*, b*, ∆ *E* , TP, CR, OP, and ∆ *E*_diff_ . Thus, the current investigation supports rejection of the null hypothesis. A significant decrease in the L* (darker) but an increase in the b* (yellower) upon aging was indicated in this study, except for VC. A significant decrease in the a* (greener) upon aging was recorded, except for TC, RA, and MH. There was evidence of increased translucency, but it decreased in contrast upon aging, except for LA and UA. There was an increase in opalescence upon aging, except for TC, LA, and UA. The study indicated color change when the provisional restorative material was subjected to aging. This color alteration is probably related to provisional materials' structural differences and optical properties. The differences in color parameters alteration were associated with the composition and manipulation of provisional material, an imperfection in the construction of the provisional restoration, and the service duration of restoration. 7 The color alteration were noted in the L*, a*, and b* coordinates, which are specific to the type of provisional material. Except for MH, the PMMA provisional restorative materials demonstrated higher color stability than bis-acryl. The CAD-CAM provisional restorative materials exhibited superior color stability than bis-acryl and heat-polymerized PMMA. The study was supported by other previous studies. 21 25 The proprietary differences in chemical compositions, for example, dissemination of particles, stability of pigment, polarity of monomers, and efficacy of resin initiator, probably impact the capability of resin polymerization, liquid absorption, and subsequently color sustainability of provisional restoration. It was reported that the polymer mixtures might be detrimental to the color sustainability of provisional restoration, deliberating that most bis-acryl resin composites denote more polarity than resin acrylic polymers. These chemical compositions encourage the affinity of bis-acryl resin composites for polarized molecules of the liquid. Therefore, more engagement of ingredients that impede the color sustainability of the provisional restorations was detected. 3 Most bis-acryl resins possess more polarity than PMMA resins and hence have a more considerable affinity with water and other polarity fluids. This probably explains the reason for the more remarkable color changes with bis-acryl resins. Moreover, the PMMA-based material has a higher homogeneous composition. Therefore, the liquid absorbing capability is probably less than bis-acryl resin, thus reducing a direct influence on the color alteration of PMMA polymer. Moreover, with the heterogeneity of bis-acryl polymer, the capability of liquid infiltration into the center of the tiny particles of material probably increases, producing many opportunities for color alteration. 22 The study revealed that the color alteration of heat-polymerized PMMA was greater than autopolymerized PMMA, CAD-CAM-based PMMA, and bis-acryl resin, except for PA. This is probably related to the fabrication porosity during processing heat-polymerized PMMA that resulted in the liquid infiltration into the material during the polymerization process and caused higher color alteration upon aging. 1 27 The differences in chemical composition may explain why temporary restorative materials demonstrate the variation in color changes and seem specific for each material. 1 Furthermore, it has primarily excellent distribution of particles in combination with different sizes of powder that permit a high-density packing of particles. This phenomenon may induce surface smoothness, which directly affects color stability.

In the CIE L*a*b* color system, the amount of color alteration between two situations is specified by ∆ *E*_diff_ . The National Bureau of Standards (NBS) introduced a sophisticated assessment of color appearance differences by NBS-ranging units. 18 20 27 . The values of ∆ *E*_diff_ between 0 and 1.5 represent extremely slight changes but imperceivable. The values of ∆ *E*_diff_ between 1.5 and 3.0 represent the perceivable color difference. The values of ∆ *E*_diff_ in the range of 3.0 to 6.0 represent an extremely marked change. The values of ∆ *E*_diff_ in the range of 6.0 to 12.0 represent marked changes. The values of ∆ *E*_diff_ beyond 12.0 indicate very much changed to other colors. The color difference values of this study were transformed into NBS-ranging units to extrapolate for clinical comparison. The color alteration of ∆ *E*_diff_ < 1 .5 was described as a discernible limit, and ∆ *E*_diff_ = 3.0 was an acceptable limit. Therefore, the color alteration for this study was perceivable but remained acceptable except for PA and MH. The PA and MH provisional materials indicated marked discoloration upon aging. Previous studies described the clinically acceptable limit of color appearance difference with human observation as ∆ *E*_diff_ = 3.7. 3 18 This experiment detected that the PA and MH groups increased darker, yellow, and red after aging.

In the previous study, TP value was influenced by the shade and types of materials. 17 The TP value of bleached shade composite resin varied from 2.0 to 7.1. 17 In this study, the translucency was also influenced by aging, types of material, and their interactions. TP values of provisional restoration varied from 1.82 to 4.40 and 2.17 to 5.89 for nonaged and aged groups, respectively. This is possibly related to the difference in resin matrix as well as the size, amount, and distribution of fillers. Moreover, a decrease in translucency can cause a degree of polymerization of the resin matrix as the increasing difference of refractive index between inorganic filler and resin matrix upon increasing resin polymerization. 16 17 This could be explained why bis-acryl resin has a higher degree of translucency change. The criterion for evaluating translucency changes was established to see whether the differences were perceivable by the naked eye. Differences in translucency value (ΔTP) of 2 and CR value (ΔCR) of 0.07 were regarded as the perceivable limit. 17 In this study, the TP and CR of all materials are considered acceptable as no group exceeded TP and CR values of 2 and 0.07, respectively. The previous study reported that the average opalescent index of human enamel was 22.9. 16 In this study, the OP of temporary restorative materials, measuring 2 mm in thickness, was between 1.69 to 3.97 and 1.97 to 5.54 for aging and NG intervention, respectively. This is probably related to the different types of polymeric materials and the thickness of samples used in the study that exhibit less opalescent than the hydroxyapatite as the principal component in the enamel. 16 However, it seems to increase in opalescent as the provisional restorative materials experienced an accelerated aging process.

Generally, the light could transmit, reflect, absorb, and scatter through the material. However, when light encounters material surface and is obstructed by inclusions such as pigment, void, or opacifiers, it will spread in a different direction and decrease translucency. 7 Translucency of each material involves reflective index and particle size. In this study, the degree of material reflection depended on the density and diffusion of filler particles. The area of dense filler particles demonstrated higher light scattering, decreasing transparency value. The CAD-CAM PMMA revealed the filler particles of 100 to 200 µm dissipated in the resin matrix. The heat-polymerized PMMA demonstrated denser filler particles of size 1 to 5 µm, whereas the autopolymerized PMMA revealed unorganized fillers distribution of size 1 to 200 µm. This could be explained by the difference in the translucence of different provisional materials tested. This study explained the optical properties of various types of provisional restorative materials in terms of color characteristics and color appearance alteration upon aging. The knowledge of optical properties could lead to more understanding and benefits in material selection. However, it should be mentioned that this study's limitation was that the material's thickness and color affect these properties. 25 26 Various initial colors of material could lead to more color sensitivity measured simultaneously. Accordingly, further study should compare various thicknesses and shades among different types of temporary restorative material upon aging.

## Conclusion

The present study concluded that the color appearance of provisional restorative materials was influenced by aging. Aging affects the color appearance, lightness, chromaticity, translucency, contrast, and opalescence of provisional materials to a different degree related to the type of materials. This resulted in various color alterations of provisional materials upon aging. The lightness of provisional materials tends to reduce and exhibit a darker appearance, while chromaticity seems to shift to red-yellow upon aging. The color stability of CAD-CAM-based PMMA and autopolymerized PMMA materials was better than bis-acryl provisional resin and heat-polymerized PMMA. Nevertheless, color appearance alteration of tested provisional materials upon aging was a perceivable limit that indicated clinically acceptable values, except for PA and MH. The alterations in translucency, CR, and opalescence of all tested provisional materials upon aging were within the perceivable limit. The study suggested that the selection of CAD-CAM-based PMMA or autopolymerized PMMA provisional materials for oral rehabilitation treatment was better than bis-acryl provisional resin and heat-polymerized PMMA in terms of color appearance stability.
